# Performance of shear-waves elastography in the non-invasive assessment of thyroid stiffness in patients with viral hepatitis

**DOI:** 10.1186/1471-2334-14-S7-P54

**Published:** 2014-10-15

**Authors:** Oana Săndulescu, Anca Streinu-Cercel, Monica Andreea Stoica, Daniela Manolache, Adrian Streinu-Cercel

**Affiliations:** 1 Carol Davila University of Medicine and Pharmacy, Bucharest, Romania; 2 National Institute for Infectious Diseases "Prof. Dr. Matei Balş", Bucharest, Romania

## Background

In chronic HCV infection, high rates of viral replication are often associated with progressive liver fibrosis. As HCV has also been shown to replicate in other organs, such as the thyroid [1], we have performed a pilot study to assess thyroid stiffness through non-invasive elastography.

## Methods

One trained operator performed shear-waves elastography (SWE) of the liver and thyroid in patients with chronic HCV infection, using Aixplorer (SuperSonic Imagine, Aix-en-Provence, France).

## Results

We assessed 21 patients with chronic HCV infection, with a male-to-female ratio of 0.6:1. The mean age was 51.4±11.9 years. The mean duration of HCV infection was 5.2±5.5 years (range: 0-20 years). The predominant HCV genotype was 1b (in 16 patients – data not available for 5 patients), and the predominant IL28-B genotype was CT (9 patients), followed by TT (7 patients) and CC (3 patients) – IL28-B data were not available for 2 patients.

Most (17, 81.0%) of the patients had received prior anti-HCV therapy with peg-interferon+ribavirin (PR) or direct-acting antivirals (DAA)-based treatment, and 13 of them displayed SVR (12 with DAA-based therapy and one with PR – the patient had an IL28-B genotype CC), while 4 displayed non-response to PR (IL28-B genotypes CT and TT).

Eight of the patients (38.1%) had a previously diagnosed thyroid dysfunction, and 4 of them (19.0%) were under thyroid substitution treatment at the time of evaluation. Five of the patients (23.8%) presented thyroid nodules on ultrasound examination.

The mean liver SWE was 9.6±4.2 kPa and the mean thyroid SWE was 25.1±10.4 kPa overall, and 26.2±11.3 kPa for the left thyroid lobe and 24.7±13.0 kPa for the right thyroid lobe.

## Conclusion

This pilot study warrants further dynamic assessment of liver and thyroid stiffness in patients with chronic HCV infection, on larger study groups. To our knowledge, this is the first such study on thyroid stiffness in HCV-infected patients.
